# Subchondral raft construction with locking plates for the treatment of Schatzker type II fractures

**DOI:** 10.1590/1413-785220172503153742

**Published:** 2017

**Authors:** Cemil Kayali, Caner Citak, Taskin Altay, Zafer Kement

**Affiliations:** 1 Bozyaka Education and Teaching Hospital Orthopaedics and Traumatology Clinics, Karabaglar Izmir, Turkey

**Keywords:** Tibial fractures/classification, Fracture fixation, internal, Bone screws, Treatment outcome

## Abstract

**OBJECTIVES:**

To evaluate the functional and radiological results of Schatzker type II fractures treated via subchondral raft screws combined with locking plates.

**METHODS:**

Twenty-four individuals were enrolled in this study between 2010 and 2014. The depressed joint line was elevated and the defect was filled with allograft. Next, two or three subchondral screws were placed in combination with a locking plate. At the last follow-up, clinical and radiological data were recorded.

**RESULTS:**

The mean follow-up period was 21.4 months (12-39). The mean Knee Society Score (KSS) and Rasmussen clinical scores were 91.5 (range, 77-100) and 16.75 (range, 14-18), respectively. The mean Rasmussen radiological score was 27.9 (range, 24-30) during the follow-up. There was no statistically significant difference between injured and non-injured sides with respect to the mechanical axis, the proximal medial tibial angle, and tibial slope. In addition, arthritis showed no difference on the non-injured side, although follow-up was short.

**CONCLUSIONS:**

The periarticular raft construction combined with the locking plate helps surgeon to maintain the anatomic line of the joint and the mechanical axis obtained during the surgery. Secondary arthritis seems to be major complication after fractures of the tibial plateau, although the functional results were satisfactory. ***Level of Evidence IV, Case Series.***

## RESUMO

**OBJETIVOS:**

Avaliar os resultados funcionais e radiológicos das fraturas de Schatzker tipo II tratadas com parafusos de plataforma subcondral em combinação com placas de trava.

**MÉTODOS:**

Vinte e quatro indivíduos foram selecionados para este estudo entre 2010 e 2014. A linha articular deprimida foi elevada e o defeito foi preenchido com aloenxerto. A seguir, dois ou três parafusos subcondrais foram colocados em combinação com uma placa de trava. No último acompanhamento, foram registrados os dados clínicos e radiológicos.

**RESULTADOS:**

O período médio de acompanhamento foi 21,4 meses (12-39). A média do Knee Society Score (KSS) e dos escores clínicos de Rasmussen foram 91,5 (faixa, 77-100) e 16,75 (faixa, 14-18), respectivamente. A média do escore radiológico de Rasmussen foi 27,9 (faixa, 24-30) durante o acompanhamento. Não houve diferença estatisticamente significante entre o lado com lesão e sem lesão, com relação ao eixo mecânico, ao ângulo medial proximal da tíbia e à inclinação tibial. Além disso, a artrite não apresentou diferença no lado sem lesão, embora o acompanhamento tenha sido curto.

**CONCLUSÕES:**

A construção de plataforma periarticular combinada com placa de trava ajuda o cirurgião a manter a linha anatômica da articulação e o eixo mecânico obtido durante a cirurgia. A artrite secundária parece ser uma complicação importante depois de fraturas do platô tibial, embora os resultados funcionais sejam satisfatórios. ***Nível de Evidência IV, Série de Casos.***

## INTRODUCTION

Split-depression tibial plateau fractures are the most commonly seen tibial injuries, accounting for 25-33% of tibial plateau fractures.^1^ Over the past three decades, many surgical techniques to treat this injury have been published; minimally invasive methods such as arthroscopy and/or C-armed fluoroscopy assisted osteosynthesis or traditional methods have been successfully used by many physicians.^2^ ^-^ ^5^

However, Schatzker type II fractures with comminuted osteochondral fragments are at the point of joint line collapse. Consequently, many authors have performed biomechanical studies to evaluate postoperative reduction loss. Subchondral raft construction is one popular methods under investigation for this purpose.^6^ ^-^ ^12^

In this study, we aimed to evaluate the clinical and radiological results of Schatzker type II fractures treated using subchondral raft screws combined with anatomical lateral plateau locking plates.

## PATIENTS AND METHODS

**Figure 1:**
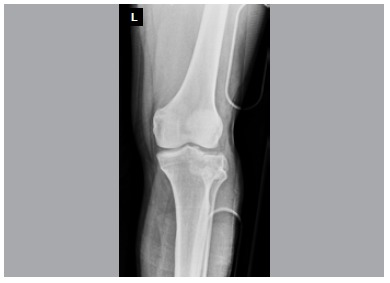
Male, 47 years, preoperative AP X-ray.

In this retrospective study, 24 consecutive Schatzker type II tibial plateau fractures treated over a three-year period were reviewed. The series consisted of only isolated tibial plateau fractures confirmed by plain x-rays and computerized tomography. (Figures 1, ^2^, ^3^A, ^3^B, ^3^C) Patients with other associated injuries, pathologic fractures, or younger than 18 years were excluded to create a uniform group. 

**Figure 2:**
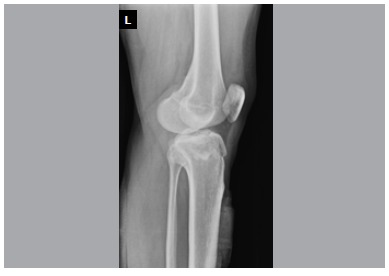
Preoperative lateral X-ray.

**Figure 3:**
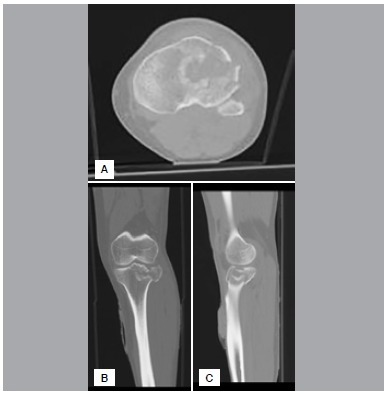
(A) Preoperative horizontal CT view; (B) preoperative frontal CT view; (C) preoperative sagittal CT view.

The sample was comprised of 19 male and 5 female patients with an average age of 45 years (range, 20-69 years). The etiology of injuries was as follows; 16 motor vehicle collisions and 8 falls from height. Mean time from hospitalization to operation was 4 days (range, 2-10 days). All patients signed an informed consent form. Because this is a retrospective study, approval was not sought from the institutional review board. 

An anterolateral incision was made over the proximal tibia, using a pneumatic tourniquet and spinal anesthesia. After the anterolateral part of the proximal tibia was exposed, fracture line was made more visible using the open book maneuver. The depressed joint line was elevated, the defect was filled with allograft and joint line congruency was observed via the submeniscal approach. Then 2 or 3 5 mm locking screws for raft construction were placed through the anatomic lateral locking plate. After C-arm fluoroscopy control, the last screws were placed and osteosynthesis was completed. Active and passive rehabilitation was begun after suction drains were removed. Partial and full weight bearing were encouraged at 6 and 10 weeks, respectively, at the outpatient clinic. 

**Figure 4:**
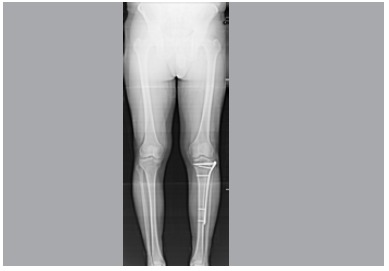
Ortho roentgenogram at last visit.

The Knee Society and Rasmussen clinical scores were used for functional evaluation.^13^ ^,^ ^14^The Rasmussen radiological score, tibio-femoral anatomical angle, proximal tibial medial angle and tibial slope were used for radiological evaluation. (Figure 4) Student's t test and the Chi-squared tests were used for statistical analysis using SPSS version 20 software.

## RESULTS

The mean follow-up period was 21.4 months (range: 12-39 months). The mean Knee Society Score (KSS) and Rasmussen clinical scores were 91.5 (range: 77-100) and 16.75 (range: 14-18), respectively. Anatomical or near-anatomical joint line reconstruction was achieved in all cases but one. A 2 mm depression was measured in the postoperative x-ray in this case. The mean Rasmussen radiologic score was 27.9 (range: 24-30) at follow-up. 

The tibio-femoral anatomical axis, proximal tibial medial angle and tibial slope angles of both the injured and uninjured sides in x-rays from the last visit were measured, recorded and compared statistically to evaluate the effectiveness of subchondral raft construction. These parameters are shown in Table 1. In summary, we found no statistical difference between the values for the injured and uninjured sides (p_TFAA_=0.265, p_PTMA_=0.574, p_TSA_=0.57).

**Table 1:** The summary of demographic, functional and radiological data of the cases. DM: Diabetes Mellitus, KSS: Knee Society Score, contr: data from contralateral uninjured side, MA: Mechanical Axe ((-) means varus), TS: Tibial Slope, PTMA: Proximal Tibial Medial Angle.

Gender	Op. day	Side	Medical history	Follow up (months)	KSS	Rass. rad.	Rass. clin.	Contr. MA	MA	Contr. T. S.	T. S.	Contr. PTMA	PTMA	Contr. arthrosis	Arthrosis
M	2	L	-	12	93	29	18	-1	-1	4	4	88	88	grade 0	grade 0
M	4	R	DM	10	87	29	16	-1	-2	5	4	87	87	grade 1	grade 1
M	3	L	-	36	90	26	16	-1	-1	4	4	87.5	87.5	grade 0	grade 1
M	2	L	-	18	100	30	18	0	0	5	5	88	88	grade 1	grade 0
F	4	R	DM	19	90	24	16	-3	-4	4	4	88	88	grade 2	grade 2
M	10	R	Alcohol	18	88	26	16	-2	-2	4	4	89	89	grade 2	grade 2
M	5	R	Smoke, Alcohol	15	87	27	16	-2	-3	4	4	86.5	86	grade 0	grade 1
M	3	R	Smoke	8	87	27	18	0	0	4	4	88	88	grade 2	grade 2
M	3	L	Smoke	28	93	29	18	0	0	5	5	86.5	86.5	grade 0	grade 0
M	2	R	Smoke, Alcohol	28	93	30	18	-1	-1	5	5	87,5	87.5	grade 1	grade 0
M	6	R	Smoke	26	90	29	18	-2	-3	5	5	87	87	grade 1	grade 1
M	4	L	-	30	77	24	18	-1	-1	4	4	86.5	86.5	grade 0	grade 1
M	5	R	-	26	93	30	18	0	0	6	6	87	87	grade 1	grade 1
M	5	L	-	24	90	26	14	-1	-1	5	5	86	86	grade 1	grade 2
M	4	L	Alcohol	11	93	25	14	-3	-2	4	3	86	86.5	grade 2	grade 2
M	2	R	Smoke	28	97	28	18	0	0	4	4	87	87	grade 0	grade 0
M	4	R	-	30	93	29	14	0	1	5	5	88	88	grade 1	grade 1
M	2	R	-	36	100	30	18	-1	-1	5	5	88.5	88.5	grade 0	grade 0
F	2	L	-	24	95	29	18	-3	-3	4	4	88	88	grade 1	grade 1
M	5	L	Smoke	21	90	26	18	-1	-1	4	4	87	87	grade 1	grade 1
F	3	L	Smoke	18	93	29	18	-1	-1	6	6	88	88	grade 2	grade 1
M	3	L	Smoke, Alcohol	18	91	29	14	-2	-3	4	5	86.5	86	grade 0	grade 1
M	5	L	-	7	93	29	18	-3	-3	3	3	87	87	grade 2	grade 1
F	7	R	DM	24	93	30	14	-3	-3	5	5	88	88	grade 1	grade 1

The range of motion (ROM) for the knee joint for both injured and uninjured sides was measured using a goniometer at the last visit. The mean ROM for the injured and uninjured sides were 133.75° (range: 120°-145°) and 137° (range: 130°-145°), respectively. There was no significant difference with respect to knee joint ROM at last visit (p=0.121).

Tibio-femoral osteoarthritis was defined using Ahlbäck classification criteria as stage 0: no radiographic sign of arthritis; stage I: narrowing of the joint space (JSN) (with or without subchondral sclerosis). JSN is defined by a space inferior to 3 mm, or inferior to the half of the space in the other compartment (or in the homologous compartment of the other knee); stage II: obliteration of the joint space; stage III: bone defect/loss <5 mm; stage IV: bone defect/loss 5-10 mm; and stage V: bone defect/loss >10 mm, often with subluxation and arthritis of the other compartment.^15^ There were 6 cases with grade 0, 13 cases with grade 1 and 5 cases with grade 2 arthritis. For comparison we evaluated the degree of arthritis in the contralateral uninjured knee. There were 10 cases with grade 0, 10 cases with grade 1 and 4 cases with grade 2 arthritis. When these groups were analyzed statistically using the greater contingency table (chi-squared test) no significant difference was observed (p=0.471). No severe osteoarthritis was seen. 

No superficial or deep infection was observed, no peroneal paralysis was recorded before or after the procedure and no hardware was removed from any patient. All patients except one returned to their pre-injury level of activity, work, or recreational activities; in this case, a 33 year old male moved from heavy labor to a desk job. His functional status was good according to KSS and Rasmussen criteria.

## DISCUSSION

Schatzker type II fractures are the most common tibial plateau injuries resulting from axial and bending forces. Type II fractures usually contain more than 1 fracture fragment because the lateral plateau is convex to the femur and features a substantial proportion of cancellous bone. Articular surface depression is widely seen in older patients because of osteoporosis. Consequently, both minimally invasive and conventional approaches are used to treat type II fractures.^1^ ^,^ ^9^

Cross et al. described the debate on ideal internal fixation for preventing subsequent loss of reduction during postoperative rehabilitation. Adequate maintenance in the postoperative period is important to avoid this outcome, due to the risk of posttraumatic arthritis. The subchondral raft technique is a well-known method to resist depression and loss of reduction and can be performed using a Kirschner wire, lag screw, conventional screw and locking screw either through the plate or individually.^10^

Raft construction has also been addressed by other authors. Cole reported that comminuted, unstable areas could be supported in their reduced position by placing a raft of parallel smaller-diameter screws close below and parallel to the articular surface. After elevation and support using a bone void filler, fixation of the lateral cortex is then achieved with a buttress plate or periarticular "raft" plate. This author recommended that the subchondral raft of screws be placed through the plate so the screws are fixed laterally at the plate and medially in the intact medial column of bone.^16^

Karunakar et al. compared biomechanical characteristics of 4 fixation options: the L-buttress plate, four 3.5 mm subchondral raft screws with an antiglide plate, an L-buttress plate with cancellous allograft and four 3.5 mm subchondral raft screws through a periarticular plate for type II fractures. These authors found no significant differences between these constructions, but the raft of subchondral screws demonstrated more resistance to local depression loads.^9^

In another biomechanical study, Cross et al. evaluated three different raft alternatives: raft construction outside the plate, non-locking raft screws through the plate and locking raft screws through the plate. These authors reported achieving statistically significant stability with the raft through plate over screws outside the plate. However, they did not find that locking screws were superior to non-locking screws and recommended considering raft construction through the plate versus outside the plate.^10^

In our study we applied 5 mm raft screws through the locking plate to support comminuted osteochondral fragments against collapse. The radiological and functional results are promising. Among the radiological parameters (tibio-femoral anatomic angle, proximal medial tibial angle and posterior tibial slope), there was no significant difference between injured and uninjured knees at the last visit.

The functional results were all good or excellent according to KSS and Rasmussen criteria. There was no significant loss of knee ROM at the last follow-up. The only issue is the stage of arthrosis detected. Post traumatic arthrosis is the most concerning complaint after tibial plateau fractures.^17^ ^,^ ^18^

Parkkinen et al. reported that factors predicting the development of early arthritis are postoperative articular congruity and normal mechanical axis. They found valgus malalignment ≥5° and articular depression >2 mm lead to severe arthritis.^19^

Another study comprised of 109 plateau fractures after a long-term follow-up (5 to 27 years) reported that cases with malalignment exceeding 5° have more moderate to severe arthritis than cases with anatomic axes. In that study, 31% of the patients had posttraumatic arthritis.^4^

Two points are important to lower arthritis rate: obtaining the anatomic joint line and normal mechanical axis during surgery and maintaining this reduction throughout the healing period.^4^ ^,^ ^19^ In addition to all initial articular cartilage damage was accused for the development of arthritis.^20^

In our study we had 18 cases with grade 1 or 2 arthritis at last visit. However, 4 cases had grade 1 arthritis in the injured knee and grade 0 arthritis in the uninjured contralateral knee and only one case had grade 2 arthritis in the injured knee and grade 1 arthritis in the uninjured knee. Arthritis developed in 4 cases and progressed one level in one case due to a plateau fracture. At this point 21% of patients have secondary arthritis which developed at a mean of 21.4 months. 

This study poses some shortcomings, namely the fact that it was a retrospective analysis with no comparative group. Although the study group seems small, it was identical, with only Schatzker type II fractures. In addition, the same surgical team was used in all cases.

## CONCLUSION 

In conclusion, periarticular raft construction through the locking plate helps surgeons achieve and preserve the anatomic joint line and normal mechanical axis. Secondary arthritis seems to be the major complication after tibial plateau fractures although superior functional results were obtained in the short term.

## References

[B1] Zhai Q, Luo C, Zhu Y, Yao L, Hu C, Zeng B, Zhang C (2013). Morphological characteristics of split-depression fractures of the lateral tibial plateau (Schatzker type II): a computer-tomography-based study. Int Orthop.

[B2] Raza H, Hashmi P, Abbas K, Hafeez K (2012). Minimally invasive plate osteosynthesis for tibial plateau fractures. J Ort hop Surg (Hong Kong).

[B3] Kayali C, Oztürk H, Altay T, Reisoglu A, Agus H (2008). Arthroscopically assistedpercutaneous osteosynthesis of lateral tibial plateau fractures. Can J Surg.

[B4] Rademakers MV, Kerkhoffs GM, Sierevelt IN, Raaymakers EL, Marti RK (2007). Operative treatment of 109 tibial plateau fractures: five - to 27-year follow-up results. J Orthop Trauma.

[B5] Timmers TK, van der Ven DJ, de Vries LS, van Olden GD (2014). Functional outcome after tibial plateau fracture osteosynthesis: a mean follow-up of 6 years. Knee.

[B6] Cooper HJ, Kummer FJ, Egol KA, Koval KJ (2002). The effect of screw type on thefixation of depressed fragments in tibial plateau fractures. Bull Hosp Jt Dis.

[B7] Patil S, Mahon A, Green S, McMurtry I, Port A (2006). A biomechanical study comparinga raft of 3.5 mm cortical screws with 6.5 mm cancellous screws in depressed tibial plateau fractures. Knee.

[B8] Westmoreland GL, McLaurin TM, Hutton WC (2002). Screw pullout strength: abiomechanical comparison of large-fragment and small-fragment fixation in the tibial plateau. J Orthop Trauma.

[B9] Karunakar MA, Egol KA, Peindl R, Harrow ME, Bosse MJ, Kellam JF (2002). Splitdepression tibial plateau fractures: a biomechanical study. J Orthop Trauma.

[B10] Levy BA, Morgan JA, Armitage BM, Cole PA, Cross WW 3rd (2013). Periarticular raft constructs and fracture stability in split-depression tibial plateau fractures. Injury.

[B11] Beris AE, Soucacos PN, Glisson RR, Seaber AV, Urbaniak JR (1996). Load tolerance of tibial plateau depressions reinforced with a cluster of K-wires. Bull Hosp Jt Dis.

[B12] Yoon YC, Oh JK, Oh CW, Sahu D, Hwang JH, Cho JW (2012). Inside out rafting K-wiretechnique for tibial plateau fractures. Arch Orthop Trauma Surg.

[B13] Insall JN, Dorr LD, Scott RD, Scott WN (1989). Rationale of the Knee Societyclinical rating system. Clin Orthop Relat Res.

[B14] Rasmussen PS (1973). Tibial condylar fractures. Impairment of knee joint stabilityas an indication for surgical treatment. J Bone Joint Surg Am.

[B15] Ahlbäck S (1968). Osteoarthrosis of the knee. A radiographic investigation. Acta Radiol Diagn (Stockh).

[B16] Cole P, Lafferty PM, Levy BA, Watson JT, Browner B, Jupiter J, Krettek C (2014). Tibial plateau fractures. Skeletal Trauma: Basic Science, Management and Reconstruction.

[B17] Mehin R, O'Brien P, Broekhuyse H, Blachut P, Guy P (2012). Endstage arthritis following tibia plateau fractures: average 10-year follow-up. Can J Surg.

[B18] Wasserstein D, Henry P, Paterson JM, Kreder HJ, Jenkinson R (2014). Risk of total knee arthroplasty after operatively treated tibial plateau fracture: a matched-population-based cohort study. J Bone Joint Surg Am.

[B19] Parkkinen M, Madanat R, Mustonen A, Koskinen SK, Paavola M, Lindahl J (2014). Factors predicting the development of early osteoarthritis following lateral tibial plateau fractures: mid-term clinical and radiographic outcomes of 73 operatively treated patients. Scand J Surg.

[B20] Manidakis N, Dosani A, Dimitriou R, Stengel D, Matthews S, Giannoudis P (2010). Tibial plateau fractures: functional outcome and incidence of osteoarthritis in 125 cases. Int Orthop.

